# Improving genomic‐informed risk assessments for dementia by combining modifiable risk factors and polygenic scores in the All of Us Research Program

**DOI:** 10.1002/alz70860_100998

**Published:** 2025-12-23

**Authors:** Clayton O Mansel, Soniya Mishra, Diego R Mazzotti, Russell H Swerdlow, Olivia J Veatch

**Affiliations:** ^1^ University of Kansas Alzheimer's Disease Research Center, Kansas City, KS, USA; ^2^ University of Kansas Medical Center, Kansas City, KS, USA; ^3^ University of Kansas Alzheimer's Disease Research Center, Fairway, KS, USA

## Abstract

**Background:**

Genomic‐informed risk assessments have the potential to improve patient outcomes for dementia through risk‐stratification and prediction of treatment‐response. Polygenic scores (PGS) offer insight into an individual's cumulative genetic risk, but their prediction accuracy is limited in individuals of non‐European ancestry and low socioeconomic status. Evidence suggests that PGS prediction accuracy is influenced by modifiable risk factors (i.e., gene by environment interactions). We therefore sought to calibrate PGS for dementia by studying its interaction with twelve well‐validated modifiable risk factors for dementia: less education, hypertension, traumatic brain injury, hearing impairment, smoking, obesity, depression, low physical activity, type II diabetes, low social contact, and excessive alcohol consumption. We hypothesize that a high PGS will be associated with increased prevalence of dementia, and that this effect will be amplified in individuals with cardiometabolic risk factors for dementia.

**Method:**

We used electronic health record (EHR) data from participants in the All of Us Research Program to identify dementia cases using a previously validated computable phenotype. We then investigated the prevalence of modifiable risk factors of interest among dementia cases. To assess feasibility, PGS were generated using whole‐genome sequencing (WGS) for 500 randomly selected cases and 500 unmatched controls. PGS were normalized and tested for association with dementia diagnosis using covariate‐adjusted logistic regression

**Result:**

Of 245,366 participants, 3,222 met dementia criteria with the following demographics: mean (SD) age of 72.2 (13.8) years, 51.1% female, 62.8% self‐identified white, and 16.5% Hispanic ethnicity. The most prevalent modifiable risk factor among dementia cases was hypertension (87%) followed by depression (63%). For participants with WGS (*n* = 2,184), 1,491 were of European ancestry, 318 African/African American, 329 American Admixed/Latino, and 46 East Asian/South Asian/Middle East. In the random sample of 1,000 individuals, a 1‐standard deviation increase in PGS was associated with a dementia diagnosis (Odds Ratio [95% CI]: 1.29 [1.05‐1.58]).

**Conclusion:**

Most individuals with dementia had co‐occurring cardiometabolic risk factors, potentially affecting genomic‐informed risk assessments. A higher PGS was associated with dementia prevalence, indicating its role in identifying at‐risk patients. Next, we will examine PGS interactions with modifiable risk factors in the full All of Us dataset.

